# *Phaseolus acutifolius* Lectin Fractions Exhibit Apoptotic Effects on Colon Cancer: Preclinical Studies Using Dimethilhydrazine or Azoxi-Methane as Cancer Induction Agents

**DOI:** 10.3390/molecules22101670

**Published:** 2017-10-08

**Authors:** Ulisses Moreno-Celis, Josué López-Martínez, Alejandro Blanco-Labra, Ricardo Cervantes-Jiménez, Laura Elena Estrada-Martínez, Alejandro Eduardo García-Pascalin, María De Jesús Guerrero-Carrillo, Adriana Jheny Rodríguez-Méndez, Carmen Mejía, Roberto Augusto Ferríz-Martínez, Teresa García-Gasca

**Affiliations:** 1 Facultad de Ciencias Naturales, Universidad Autónoma de Querétaro, Av. de las Ciencias s/n. Juriquilla, CP 76230 Querétaro, Mexico; ulisses.morenoc@gmail.com (U.M.-C.); bioxtremo@hotmail.com (J.L.-M.); ricardocervantesjimenez@gmail.com (R.C.-J.); laurel_1610@hotmail.com (L.E.E.-M.); alejandroe.garciap@gmail.com (A.E.G.-P.); fryda5263@yahoo.com.mx (M.D.J.G.-C.); maria.c.mejia@uv.es (C.M.); 2 Depto de Biotecnología y Bioquímica, CINVESTAV-Unidad Irapuato, Guanajuato CP 36821, Mexico; alejandroblancolabra@gmail.com; 3 Facultad de Medicina, Universidad Autónoma de Querétaro, Querétaro CP 76176, Mexico; adjen.rm@gmail.com

**Keywords:** colon cancer, apoptosis, lectins, Tepary bean, *Phaseolus acutifolius*

## Abstract

*Phaseolus acutifolius* (Tepary bean) lectins have been studied as cytotoxic molecules on colon cancer cells. The toxicological profile of a Tepary bean lectin fraction (TBLF) has shown low toxicity in experimental animals; exhibiting anti-nutritional effects such as a reduction in body weight gain and a decrease in food intake when using a dose of 50 mg/kg on alternate days for six weeks. Taking this information into account, the focus of this work was to evaluate the effect of the TBLF on colon cancer using 1,2-dimethylhydrazine (DMH) or azoxy-methane/dextran sodium sulfate (AOM/DSS) as colon cancer inductors. Rats were treated with DMH or AOM/DSS and then administered with TBFL (50 mg/kg) for six weeks. TBLF significantly decreased early tumorigenesis triggered by DMH by 70%, but without any evidence of an apoptotic effect. In an independent experiment, AOM/DSS was used to generate aberrant cryptic foci, which decreased by 50% after TBLF treatment. TBLF exhibited antiproliferative and proapoptotic effects related to a decrease of the signal transduction pathway protein Akt in its activated form and an increase of caspase 3 activity, but not to p53 activation. Further studies will deepen our knowledge of specific apoptosis pathways and cellular stress processes such as oxidative damage.

## 1. Introduction

Plant lectins are proteins that are able to bind to free or cell membrane carbohydrates, showing a high specificity for the recognition of surface glycosylations. Given these properties, they have been used for several disease diagnoses and as potential therapeutic agents, including against cancer [1,2,3]. Several studies have shown that traditional-use plant lectins exhibit biological effects such as cell agglutination, reactive oxygen species generation, and mitogenic or cytotoxic effects on different cells as a function of concentration [3]. Lectins from edible and non-comestible plants, such as banana and ricin, have shown different effects that are related to different molecular mechanisms. The wide-range recognition ability of lectins is related to their specificity for membrane carbohydrates, therefore it is difficult to generalize the effects of these molecules [4,5].

Lectins can exhibit mitogenic effects, for example, peanut lectins have shown effects on cell proliferation [6,7], and others such as mistletoe (*Viscus album*) have mitogenic effects on immune cells [3]. On the other hand, soybean and other leguminous lectins have shown anticancer potential by promoting blockage of survival signaling pathways or inducing apoptosis by increasing reactive oxygen species (ROS), and/or caspase activity [8,9]. Changes in glycocalix are related to cancer progression due to their relationship with cell migration, invasion, evasion of the immune system, and metastasis [10,11,12,13], therefore, lectins have been widely studied for their anticancer effects [14], cell proliferation [15,16,17,18], and the increase in cell death by apoptosis and autophagy [12,13]. Some lectins can affect signal transduction pathways, such as mistletoe lectins that inhibit Akt phosphorylation [19] or *Polygonatum cyrtonema* lectins, which exhibit effects on the Ras-Raf and PI3K-Akt pathways [20] and induce apoptosis by deregulation of the ROS-p38-p53- signal pathway [21]. However, some lectins can induce apoptosis by independent p53 pathways [22].

*Phaseolus acutifolius* (Tepary bean) lectins have been studied recently because of their differential cytotoxic effects on cancer cell lines, particularly their specificity for colon cancer cells [17,23]. Toxic effects by intraperitoneal [24] and intra-gastric [25] routes have been tested and a Tepary bean lectin fraction (TBLF) showed good tolerability when administered in a dose of 50 mg/body weight kg, on alternate days for six weeks in Sprague Dawley rats by intra-gastric cannula. The TBLF was characterized, two lectins with no affinity for fetuin were sequenced and a differential biological activity was found [26]. Here, we demonstrate that the lectins present in TBLF are the only ones responsible for the cytotoxic effect and the evaluation of the intra-gastric administration of TBLF on chemical-induced colon cancer in Sprague Dawley rats using two cancer inductors, 1,2-dimetilhydrazine (DMH) and azoxi-methane/dextran sodium sulfate (AOM/DSS), is presented.

## 2. Results and Discussion

**Figure 1:**
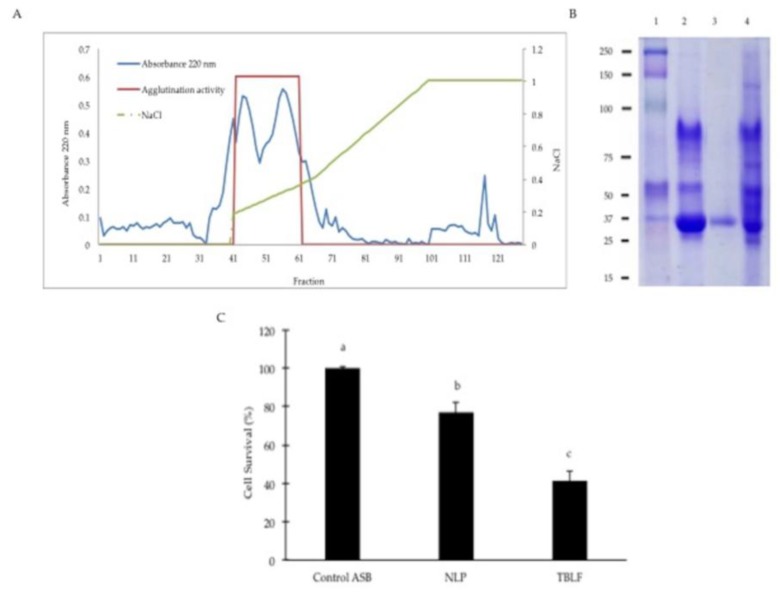
Cytotoxic effect of the non-lectin protein contained in the Tepary bean lectin fraction (TBLF). (**A**) TBLF was subjected to an ion exchange chromatography were non-lectin protein (NLP) and lectins (fractions with agglutination activity) were separated. (**B**) Electrophoretic profile of NLP and lectins. (1) Molecular weight marker, (2) TBLF, (3) Lectin pool, (4) Non-lectin protein pool. (**C**) Cytotoxic effect of NLP compared to TBLF. Small letters show significant difference by one-way ANOVA for the cytotoxic effect (Tukey, *p* ≤ 0.05).

In previous work, we showed that protein inhibitors from Tepary beans did not exhibit cytotoxic effects but the TBLF is able to induce cell death in a differential manner on different cancer cells [11]. Prior to testing the effect of TBLF against colon cancer in rats, we confirmed that the non-lectin proteins (NLP) contained in the TBLF were not responsible for the cytotoxic effect. The ion exchange chromatogram obtained from the TBLF is shown in Figure 1. All the fractions with no agglutination activity were labelled as non-lectin protein (NLP); fractions with agglutination activity were pooled and labelled as lectin. The electrophoretic profile shows that the characteristic lectin band was separated from all the protein and the separation method showed good results in reproducibility. The lectin fraction was insufficient to perform the cytotoxic analysis but it was possible to test the effect of the NLP on cell proliferation. As cell harvesting was not achieved by using trypsin, we used an image analyzer to measure the cytotoxicity of the NLP. Image variables showed good correlation with cell proliferation by direct counting: cell circularity (−0.972, *p* < 0.001), Feret’s diameter (0.854, *p* = 0.015) and cell perimeter (0.899, *p* = 0.006) but the cell area did not show correlation (0.578, *p* = 0.174). Taking this, we used only the significant image parameters to determine that lectins are the molecules mainly responsible for the cytotoxic effect since NLP exhibited a low cytotoxicity when compared with the TBLF (Figure 1C).

**Figure 2:**
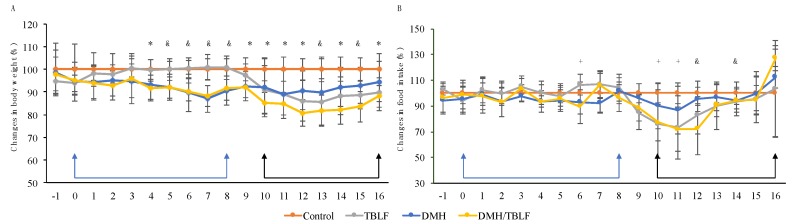
Body weight and food intake changes for the 1,2-dimethylhydrazine (DMH) experiment. Rats were administered DMH for eight weeks for the development of cancer lesions (blue arrows), followed by intra-gastric administration of TBLF (50 mg/kg) on alternate days for six weeks (black arrows). (**A**) Weekly body weight monitoring with respect to the control group. (**B**) Average of food intake changes per week with respect to the control group. A one-way ANOVA by Dunnett post hoc showed significant difference *p* ≤ 0.05 (*), *p* ≤ 0.01 (+) and *p* ≤ 0.001 (&). TBL provoked a decrease in weight gain that affected also the combined DMH/TBLF treatment. Food intake decreased through TBLF administration but a final recovery was observed.

Two in vivo experiments were performed to study the effects of TBLF treatment on induced colon cancer. The first set of experiments consisted of inducing tumors in colonic tissue using DMH. Previous work showed that TBLF did not exhibit toxic effects in Sprague Dawley rats when orally administered in a dose of 50 mg/body weight kg; the only adverse effect observed until then was a 10% reduction of body weight gain [25]. Our results showed that TBLF caused a statistically significant decrease in body weight gain of about 10% and DMH administration caused a decrease of 5% (*p* < 0.0001, *F* = 21.68 and df = 71). The DMH/TBLF treated group showed a body weight gain reduction of up to 20%, with a recovery of 10% at the end of the treatment (Figure 2A). These indicated a slight detrimental effect of DMH on body weight gain, but as TBLF naturally provokes a lessening in this parameter, it appeared that the co-administration of DMH/TBLF had a potentiated effect through the treatment that concluded with a partial recovery of body weight. In terms of food intake, there were variations in food consumption throughout the experiment; a decrease of 25% for TBLF and DMH/TBLF treatments even showed a complete recovery at the end of the experiment (Figure 2B) but there is no statistically significant difference (*p* = 0.4765, *F* = 0.8383 and df = 71). These results suggested that the detrimental effects on body weight gain and food intake were mainly related to the TBLF effect as an anti-nutritional factor. Besides anti-nutritional effects, TBLF could also exhibit effects on the immune system by mainly increasing granulocytes, and decreasing lymphocytes without effects on hepatic, renal or pancreatic markers [25], suggesting no toxic effects.

**Figure 3:**
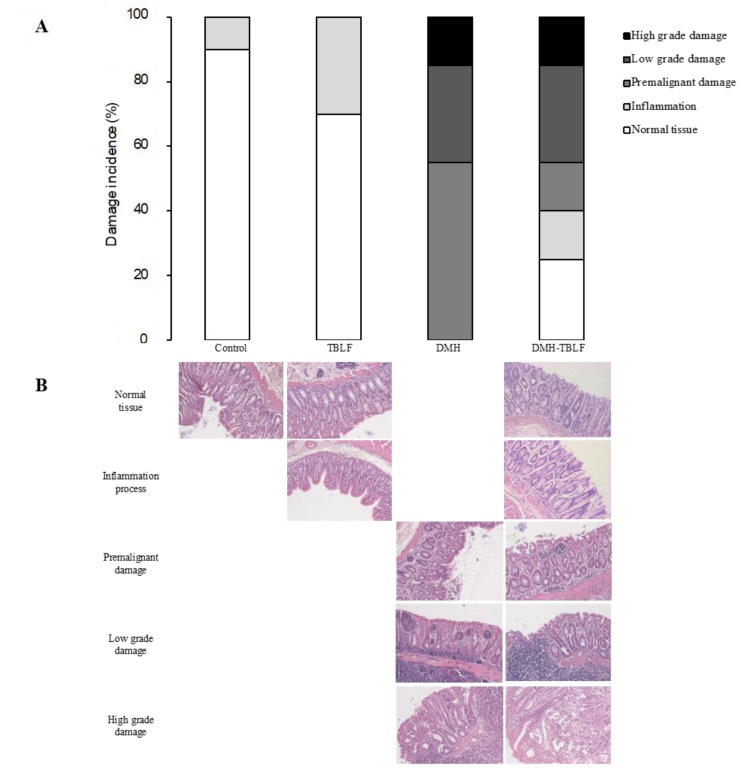
Histopathological analysis for colon lesions provoked by DMH and treated with TBLF. Rats were administered DMH for eight weeks for the development of cancer lesions followed by intra-gastric administration of TBLF (50 mg/kg) on alternate days for six weeks. (**A**) Incidence of colon tumors. (**B**) Hematoxylin-eosin histopathological analyses of colon lesions (10×). Microphotography shows the histological changes where it can be observed that tissue architecture was disrupted according to the malignancy.

Tumor induction with DMH is shown in Figure 3. Histopathological findings showed different stages of tumor progression that were classified as inflammation, premalignant damage, low-grade damage and high-grade damage [27]. TBLF increased inflammation in colon tissues, lectins are considered anti-nutritional factors [2,28] because they can resist gut digestion for several hours, or even days [25]. They bind to intestinal membrane glycoproteins or glycolipids that affect nutrient absorption [7,29], and also activate the immune response [25,30], thereby provoking inflammation. Previous studies have shown that TBLF has exhibited a resistance to gut digestion, since agglutination activity was determined in faeces for at least 72 h with immune response activation [25], suggesting that they can interact with intestinal and colon epithelia, leading to an inflammation process.

**Figure 4:**
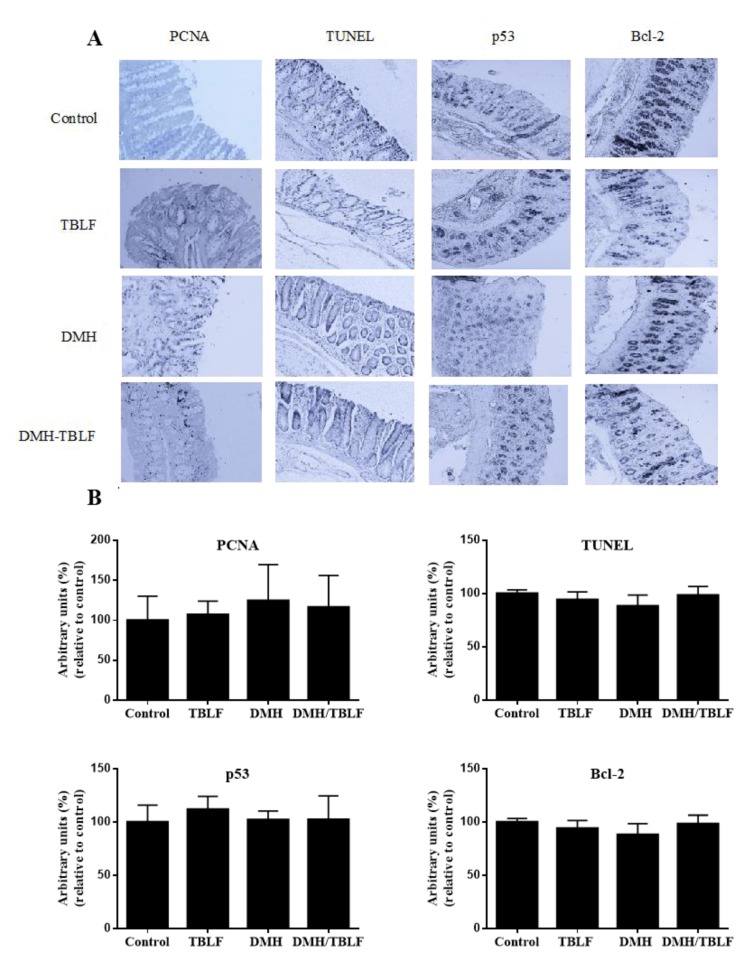
Immunohistochemical analyses of proliferation and apoptosis markers in colon lesions provoked by DMH and treated with TBLF. Rats were administered DMH for eight weeks for the development of cancer lesions followed by intra-gastric administration of TBLF (50 mg/kg) on alternate days for six weeks. (**A**) Representative micrographs of each group (10×); (**B**) Densitometry analyses of immuno-stained colon tissues. No significant difference by one-way ANOVA (Dunnett, *p* < 0.05) was found. No significant changes were observed between treatments.

DMH provokes different degrees of precancerous or cancerous injuries. Our results showed that DMH/TBLF treatment decreased 70% of premalignant lesions. Other lectins tested in different models have shown negative effects on tumorigenesis [31,32]. When proliferation and apoptotic markers were studied by immunohistochemical analyses, no differences were observed between the control rats and any of the treated groups (Figure 4). These results may be related to the tumor progression stage as the main activity of TBLF was observed in early tumorigenesis. Changes in glycocalyx are essential for lectin recognition, and it is well known that tumor progression is related to modifications in membrane glycosylation. Cancer cells display different aberrant membrane glycosylation patterns depending on the type of cancer and the tumor stage [21], therefore, TBLF was most likely unable to recognize low- and high-grade tumor cells.

**Figure 5:**
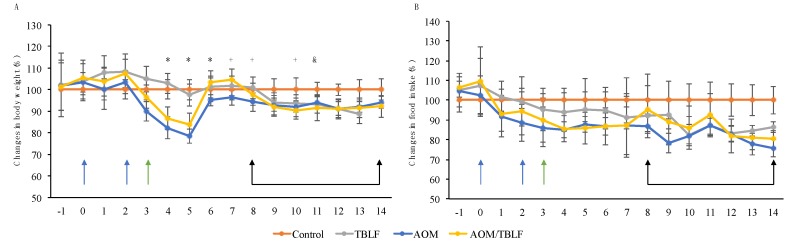
Body weight and food intake changes for the azoxy-methane/dextran sodium sulfate (AOM/DSS) experiment. Rats were intraperitoneally administered for two weeks with AOM (blue arrows) and a further week with dextran sodium sulfate (DSS) in their daily drinking water (green arrow) to develop aberrant cryptic foci (ACF). Next, they were administered TBLF (50 mg/kg) on alternate days for six weeks via intra-gastric cannula (black arrows). (**A**) Weekly body weight monitoring with respect to the control group; (**B**) Average of food intake changes per week with respect to the control group. A one-way ANOVA by Dunnett post hoc showed significant difference (*) *p* ≤ 0.05, (&) *p* ≤ 0.01 and (+) *p* ≤ 0.001. A decrease in weight gain and food intake was observed for all treatments with no recovery at the end of the experiment.

As our results showed effects of TBLF on early tumorigenesis, a second set of experiments were performed using AOM/DSS in order to induce aberrant cryptic foci (ACF). AOM/DSS, alone or in combination with TBLF, showed a negative effect on body weight by up to 25%, which recovered by up to 5% at the end of the treatment (Figure 5A); the results were statistically significant (*p* = 0.0004, *F* = 7.33, df = 67). Food intake showed significant differences in AOM/DSS and AOM/DSS-TBLF treated groups (Figure 5B), and was statistically different (*p* = 0.0001, *F* = 12.6, df = 67). TBLF showed a similar effect on body weight gain as previously observed in this experiment, and a decrease in food intake had effects related to the anti-nutritional effects of different lectins.

**Figure 6:**
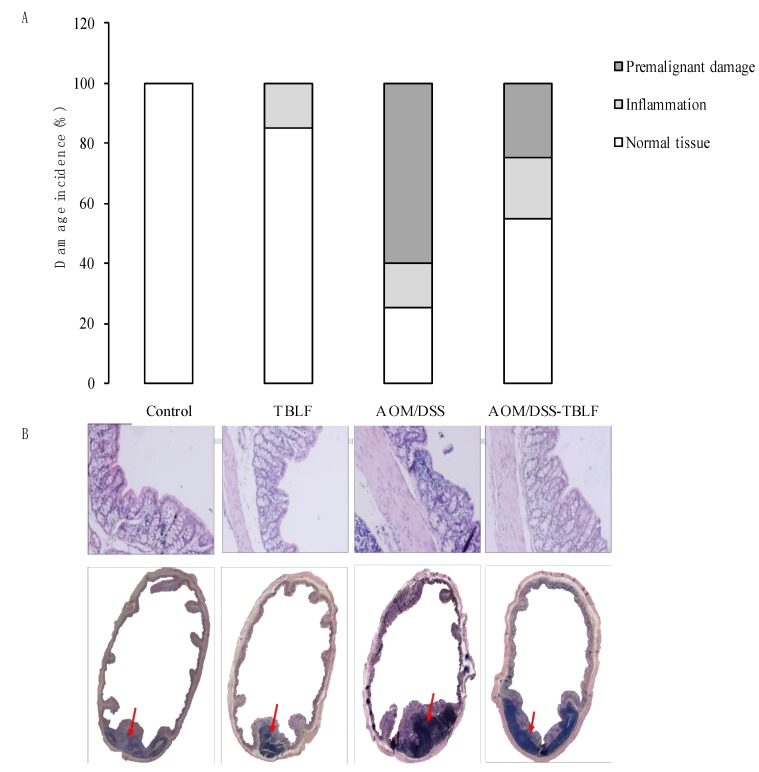
Histopathological analyses for colon lesions provoked by AOM/DSS and treated with TBLF. Rats were intraperitoneally administered for two weeks with AOM and a further two weeks with DSS in their daily drinking water to develop ACF. Next, they were administered with TBLF (50 mg/kg) on alternate days for six weeks via intra-gastric cannula. (**A**) Incidence of aberrant cryptic foci; (**B**) Hematoxylin-eosin histopathological analyses of colon lesions (10×). Red arrows show lymphocyte infiltration. Although no tumors were observed, AOM/DSS treatment showed a high disruption process that was diminished by TBLF.

Colon histopathology assays showed normal colonic tissue architecture in the control rats [27], while rats treated with the TBLF showed an atrophic effect on intestinal epithelia with decreased length and fusion of the colonic structure (Figure 6). These results were consistent with other studies, for example, Daprà et al. [23], observed that legume lectins provoked lymphocyte infiltration in the intestinal tissue as well as villi shortening and widening in trout (*Oncorhynchus mykiss*). As villi affectation by lectins can induce changes in organ architecture, this was related to the decrease in weight gain and in food consumption. The effect of AOM/DSS treatment induced inflammation in 15% and ACF in 60% of the rats’ colon tissue and disrupted the entire architecture [27]. The co-administration AOM/DSS-TBLF reduced the ACF by 50% and partially restored the tissue architecture, but atrophy was still observed. This result showed the effect of TBLF in the early stages of colon tumorigenesis; and was consistent with studies reporting anticancer effects using lectins such as Concanavalina A lectin in other animal models [4,32], and *Euchema serra* agglutinin in mice [4]. Such results agree with the fact that glycosylation changed by tumor progression affected the TBLF affinity for cancer cells in different stages.

**Figure 7:**
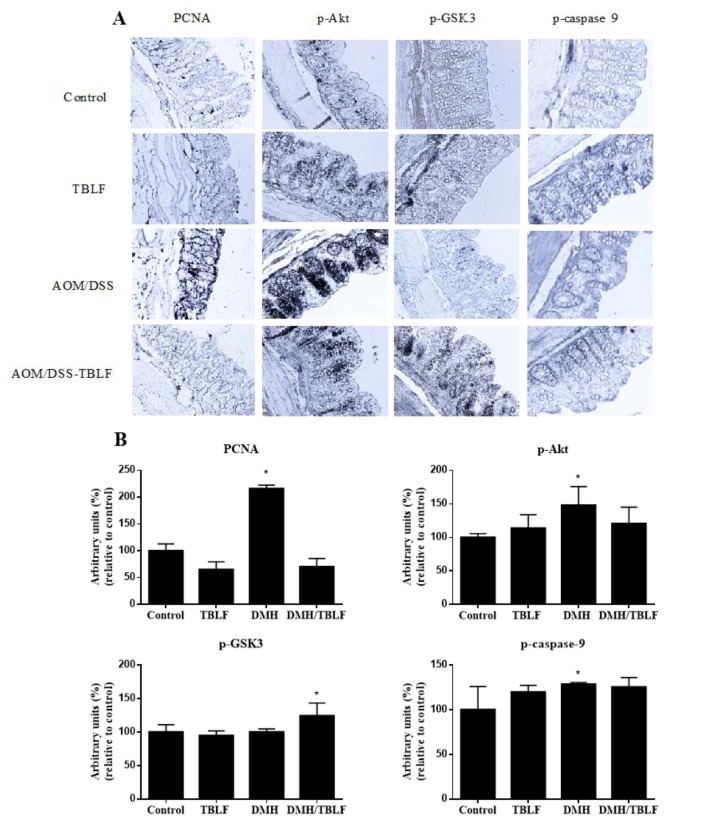
Immunohistochemical analyses of the proliferation and Akt-pathway targets in colon lesions provoked by AOM/DSS and treated with TBLF. Rats were intraperitoneally administered for two weeks with AOM and a further two weeks with DSS in their daily drinking water to develop ACF. Next, they were administered with TBLF (50 mg/kg) on alternate days for six weeks via intra-gastric cannula. (**A**) Representative micrographs of each group (10×); (**B**) Densitometry analyses of immuno-stained colon tissues. (*) Significant difference by one-way ANOVA (Dunnett *p* < 0.05).

Different studies have focused on elucidating the signaling pathways triggered by lectins, where it has been observed that lectins from different sources share some mechanisms of action on cell proliferation, survival, and apoptosis induction [33]. Immunohistochemical analysis of proliferating cell nuclear antigen (PCNA) allowed us to observe that TBLF caused a significant reduction in cell proliferation when it was administered after the carcinogen (Figure 7). These results were consistent with in vitro and in vivo studies of different lectins on cancer [7,8,32,34,35]. 

The Akt pathway was evaluated due to its participation in proliferation-apoptosis regulation and as it is commonly deregulated in colon cancer [36,37]. The Akt pathway increases cell proliferation by phosphorylating GSK-3, a blocking agent of Cyclin-d, and decreases apoptosis by phosphorylation of caspase 9. No changes between the control and TBLF treated animals were observed; however, AOM/DSS exhibited an increase in p-Akt as reported in colon cancer models [38], and also a slight increment of p-caspase 9. Nevertheless, p-GSK-3 did not show differences with respect to the control rats. These results showed the participation of the Akt pathway by AOM/DSS in colon tissue. When rats were treated with AOM/DSS-TBLF, a decrease in p-Akt was observed with respect to the AOM/DSS-treated animals, suggesting that TBLF may block the Akt pathway. Given those results, no increase in p-GSK-3 and p-caspase 9 was expected; however, p-GSK-3 increased significantly, perhaps due to the participation of other routes such as the protein kinase A (PKA) or protein kinase C (PKC) pathways [39]. 

**Figure 8:**
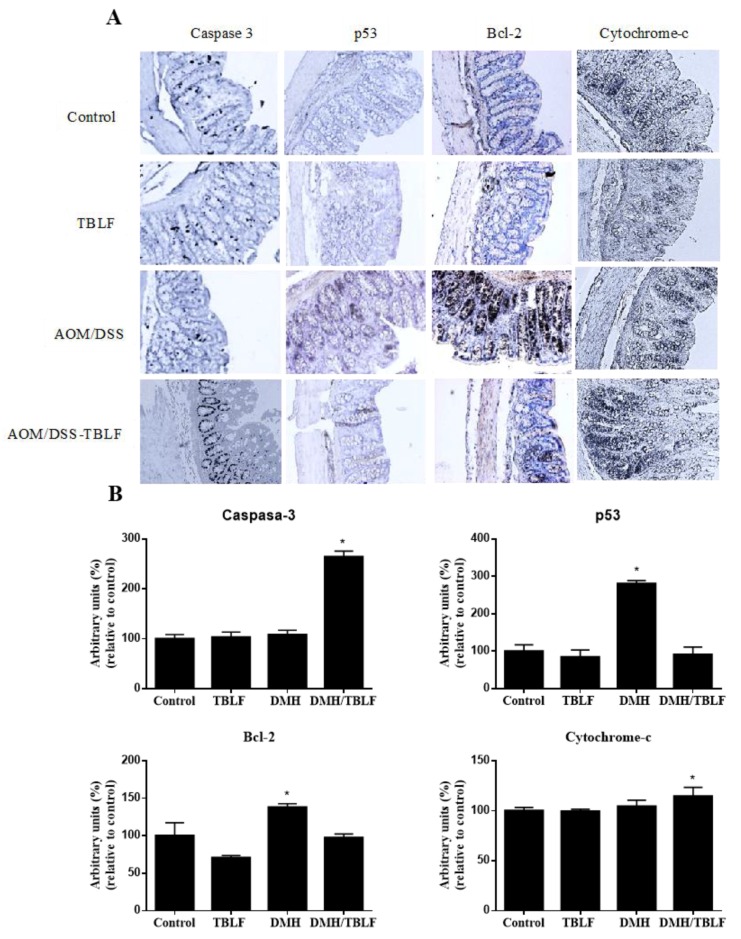
Immunohistochemical analyses of apoptosis markers in rats administered with AOM/DSS and treated with TBLF. Rats were intraperitoneally administered for two weeks with AOM and a further two weeks with DSS in their daily drinking water to develop ACF. Next, they were administered with TBLF (50 mg/kg) on alternate days for six weeks via intra-gastric cannula. (**A**) Representative micrographs of each group (10×); (**B**) Densitometry analyses of immuno-stained colon tissues. (*) Significant difference by one-way ANOVA (Dunnett *p* < 0.05).

To assess whether cell death by apoptosis induction occurred, the presence of p53, Bcl-2, caspase 3, cytochrome-c was evaluated. Neither AOM/DSS, nor TBLF alone provoked an increment of caspase 3, but the co-treatment of AOM/DSS-TBLF showed a significant increase. Furthermore, the AOM/DSS-TBLF treatment showed an increase in cytochrome-c, and a decrease in the antiapoptotic protein Bcl-2. A significant increase of p53 was observed in the AOM/DSS treatment (*p* < 0.05) (Figure 8). The specific mutations of AOM/DSS are well known, especially in KRas with high frequency [40,41], which deregulates the corresponding signaling cascade and allows sustained proliferative activity [42]. However, AOM/DSS treatment in colon cancer models was not related to mutations on the p53 gene, but an increase has been observed in the expression of this protein [38]. Studies in colitis and colon cancer models using AOM/DSS as the carcinogen have shown an increase of both the expression and amount of p53; however, no mutations have been observed [43,44]. Although the p53 protein is typically involved in repairing damage or inducing apoptosis in cells depending on DNA damage, a mechanism called apoptosis-induced proliferation (AiP) has been described [38,45] where an exacerbated apoptotic process leads to a release of mitogens that induce cell proliferation. This process has been implicated in intestine cell growth in mice, which was promoted by an increase in p53 activity [45]. In the same way, dying xenograft cells in mice treated with radiotherapy showed an increase in caspase 3 activity, which promoted tumor repopulation [46]. Therefore, induces the AiP process as part of its action mechanism in the development of colon cancer. The AOM/DSS-TBLF group showed no statistically significant differences in p53 when compared with the control group, suggesting an inhibitory effect of TBLF on AOM/DSS cancer induction.

**Figure 9:**
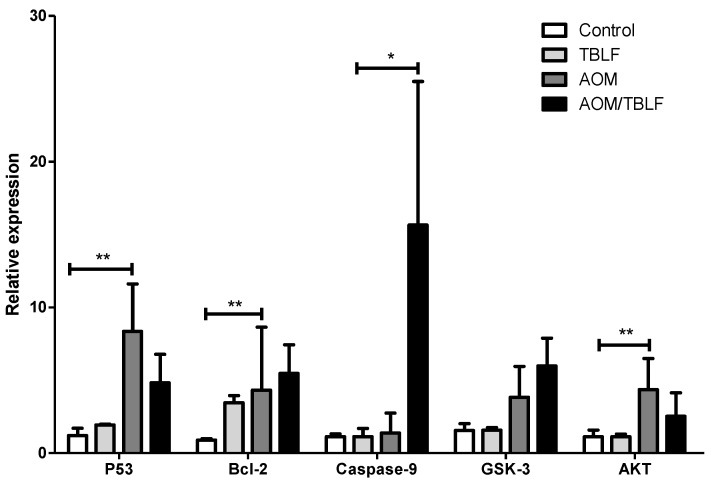
Evaluation of the gene expression of TP53, Bcl-2, caspase 9, GSK-3, and AKT in rat colon tissues administered with AOM/DSS and treated with TBLF. Rats were intraperitoneally administered for two weeks with AOM and a further two weeks with DSS in their daily drinking water to develop ACF. Next, they were administered with TBLF (50 mg/kg) on alternate days for six weeks via intra-gastric cannula. The results are shown as a relative expression to the constitutive gene HPRT and as a proportion of the control ± SD. (*) indicates significant difference *p* ≤ 0.05 and (**) indicates significant difference *p* ≤ 0.01 by one-way ANOVA with Dunnett post hoc.

In order to corroborate the immunohistochemical results, gene expression of TP53, Bcl-2, caspase 9, GSK, and Akt was determined in colonic tissues (Figure 9). A significant increase in TP53, Bcl-2, and Akt expression were observed for AOM/DSS treatment, but no differences were found for AOM/DSS-TBLF-treated rats with respect to the control. Caspase 9 gene expression was significantly increased by AOM/DSS-TBLF treatment, suggesting apoptosis induction by TBLF. These results fully agree with the immunohistochemical analyses previously described and taken together, these results suggest the independent p53 apoptotic activity of TBLF on early precancerous colonic tissue.

It has been reported that several lectins induce apoptosis through caspase activity in different cancer cell lines [47] and in some animal models [32,44]. Mistletoe lectins (*Viscum album* L. var. Coloratum) have shown anticancer effects by inducing apoptosis by caspases in melanoma [44]. *Eucheuma serra* lectins have exhibited a caspase-mediated apoptotic effect in colon adenocarcinoma cells (Colon26) and in BALB/c mice with colon cancer [8]. The Akt-mediated signaling pathway has also been studied and the results showed that lectins such as *Canavalia brasiliensis* and *Viscum album coloratum* could induce cell death by deregulating this pathway [8,19,48]. The effect of Con-A was found to be dependent on the decrease in p-Akt, an increase in cytochrome-c, and an increase in caspase 9 activity [19,43]. In contrast, Korean mistletoe lectins (VAL) have the ability to induce p53-independent apoptosis, causing a decrease in Bcl-2 levels, and an increase in cytochrome-c and telomerase inhibitory activity on hepatic carcinoma [49]. Here, our study showed for the first time that Tepary lectins were able to induce apoptosis in colon precancerous lesions by a p53-independent way, suggesting Akt pathway blockade.

## 3. Materials and Methods

### 3.1. Tepary Bean Lectin Fraction (TBLF) Extraction

Tepary bean (*Phaseolus acutifolius*) seeds were obtained from a local market in Hermosillo, Sonora, México. A sample of Tepary bean was deposited and identified in the herbarium of Dr. Jerzy Rzedowski of The Natural Sciences Faculty, Autonomous University of Querétaro, Santiago de Querétaro, Mexico. The TBLF was obtained as described previously [17,50]. In brief, ground bean seeds were degreased and an aqueous extract was obtained. A selective sequential precipitation (40–70% ammonium sulfate) was performed and the protein dialyzed and separated by molecular weight exclusion chromatography using a Sephadex G-75 column (Sigma-Aldrich Co. LLC, St. Louis, MO, USA). Absorbance at 280 nm was measured for protein profile determination using a Beckman DU-65 spectrophotometer (Beckman Coulter Inc., Brea, CA, USA), agglutinant activity was determined [51] and the samples were lyophilized and stored for further use at −20 °C. 

### 3.2. Confirmation of the Cytotoxic Role of Lectins in the TBLF

TBLF was subjected to ion exchange chromatography using an Econo Pack High Q cartridge (ECONO, Bio-Rad Laboratories, Inc., Hercules, CA, USA) at a flow rate of 1 mL per min using a NaCl (0.1 to 1.0 M). A total of 120 fractions were collected and the presence of agglutination activity was determined. Two pools were obtained, one with all the fractions with no agglutination activity (non-lectin protein, NLP) and the other one containing the lectins. The two pools were dialyzed, lyophilized and stored at −20 °C. Cytotoxicity of NLP was determined using 3T3/v-mos cells [11]. Briefly, cells were plated in 24-well plates (1 × 10^4^ cells/well) in Dulbecco’s Modified Eagle’s Medium (DMEM) with 10% foetal bovine serum (FBS). After 24 h, the medium was changed to DMEM with 2% FBS for cell cycle synchronization for a further 24 h. Subsequently, the cytotoxic effect of the NLP was determined and compared to a TBLF positive control, using a protein concentration of 0.4 mg/mL for both treatments in DMEM with 0.5% of bovine serum albumin (BSA). Cells were incubated for 8 h and the cytotoxic effect was determined by image analysis (IMAGEJ 1.50b, National Institutes of Health, Bethesda, Rockville, MD, USA) using an inverted microscope (Carl Zeiss Axiovert A1, Carl Zeiss Inc., Oberkochen, Germany) at 10× resolution to measure the following morphometric parameters: area, perimeter, Feret’s diameter and circularity. As a first step, images of the treated cells were processed converting the colored-image to an 8-bit format (grey scale image). Then, an automatic threshold by an algorithm default was applied to obtain a binary image [52]. Finally, cells were highlighted with the tool “fill” found in the same software. Correlation with cell proliferation by direct counting was determined for each morphometric parameter and for the cytotoxic evaluation only parameters with significant correlation were used. The criterion to discriminate between living cells and dead cells was based on the statistical evaluation of morphometric parameters using ANOVA, Tukey and Fisher tests (*p* ≤ 0.05) using MINITAB 17 (Minitab Inc., Harrisburg, PA, USA) with respect to control cells where a confidence interval for each significant parameter was selected: perimeter (90.0 to 96.8 µm), Feret’s diameter (32.0 to 34.0 µm) and circularity (0.31 to 0.35), cells whose parameter values did not belong to these intervals were classified as dead cells.

### 3.3. Experimental Animals and Cancer Induction

**Figure 10:**
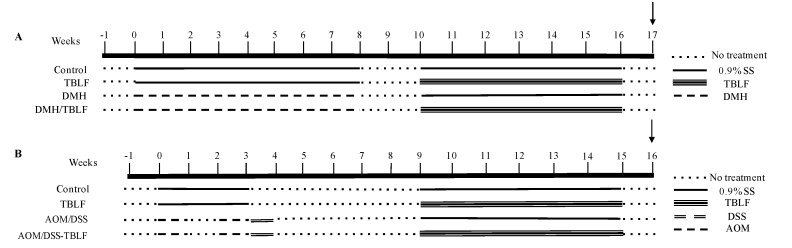
Experimental design. (**A**) DMH experiment (*n* = 10 per group). (**B**) AOM/DSS experiment (*n* = 7 per group). Two independent experiments were performed. Cancer induction with DMH was achieved after eight weeks of treatment to generate colon tumorigenesis while AOM induction took two weekly doses to generate aberrant cryptic foci. TBLF was administered for six weeks each third day and sacrificed a week later (black arrow).

Five-week-old, male Sprague Dawley rats were obtained from the Neurobiology Institute-UNAM, Juriquilla, México. The animals were maintained with food and water ad libitum, under a circadian cycle of 12 h light and 12 h dark at 25 °C. All experiments started after a week of adaptation and were conducted observing the procedures of the Official Mexican Norm [29] for the use of laboratory animals and with the approval of the Bioethics Committee of the Neurobiology Institute, UNAM, México. Two independent experiments were performed (Figure 10). The first experiment (*n* = 10 per group) induced colon tumors by subcutaneous injection of 40 mg/body weight kg of 1,2-dimethylhydrazine (DMH, D161608-100G, Sigma Aldrich, St Louis, MO, USA), in 0.5 mL of saline solution (SS, 0.9% NaCl) twice a week for eight weeks, followed by two weeks without treatment and six weeks of TBLF (50 mg/kg each third day). The second experiment (*n* = 7 per group) was designed to provoke aberrant cryptic foci (ACF) using 10 mg/body weight kg of 2-azoximethane (AOM, A5486-100MG, Sigma Aldrich, St Louis, MO, USA) in 0.5 mL of 0.9% SS by a weekly intraperitoneal injection in weeks one and three, followed by one week of 2% dextran sodium sulfate (DSS) in their daily drinking water as a cancer promoter. After five weeks without treatment, TBLF was administered in a dose of 50 mg/kg in 0.5 mL SS via intra-gastric cannula [25] on alternate days for six weeks. Weekly body weight changes and average food intake were monitored in both experiments. Euthanasia was performed by decapitation a week after the last dose of TBLF. The organs were dissected under the supervision of a veterinarian and fixed in 10% formaldehyde, and the intestines were perfused with the same solution to achieve the best preservation. Tumor classification was performed by a veterinarian pathologist [27].

### 3.4. Histopathology Analyses

The tissues were dehydrated and embedded in paraffin using a Histoquinete (Leica TP1020, Leica Camera AG, Wetzlar, Germany). Tissue cuts of 5 μm were obtained and mounted in gelatin-coated slides in hot water. Tissues were rehydrated and stained with hematoxylin and eosin, then dehydrated again using decreasing concentrations of alcohol and sealed with entellan and a coverslip. The analyses were performed under a microscope (Zeiss Axio Vert-A1, Carl Zeiss Inc., Oberkochen, Germany) using 5–10× magnification based on the morphology of normal colonic tissue. Lieberkühn crypts normally appear as simple tubular glands and can be classified according to morphological changes, inflammation process and structure loss for classifying damage as premalignant, low-grade damage, high-grade damage and neoplasia [27].

### 3.5. Immunohistochemical Analyses for Proliferation and Apoptosis

Dehydrated samples were embedded in paraffin blocks and cut into 5 μm thick positively charged Thermo Fisher slides (Thermo Fisher Scientific, Waltham, MA, USA) using a microtome and subsequently rehydrated. To unmask the epitopes, samples were placed in a water bath at 100 °C for 20 min in 0.1 M citric acid solution, pH 6.0. Endogenous peroxidases were quenched with 2% H_2_O_2_ for 30 min in phosphate buffer saline with 1% tween 20 (PBST). Blocking was performed with 1% bovine serum albumin (BSA) in PBST for 1 h and incubated with primary antibodies (PCNA, caspase 3, p53, Bcl-2, p-Akt, p-GSK3, p-caspase 9, and cytochrome-c overnight (16 h approx.) at 18 °C. Secondary antibodies were added and incubated for 2 h at 18 °C. Staining was achieved using diaminobenzidine 1:8000 *w*/*v* and H_2_O_2_ 1:2500 in phosphate buffer saline (PBS). The reaction was stopped using ddH_2_O. Hematoxylin was used as a contrast agent; samples were dehydrated and mounted for observation under a microscope. Photographs were obtained and an optical densitometry analysis was performed using Image J^®^ software (v.1.5, National Institutes of Health, Bethesda, Rockville, MD, USA). Data were reported as arbitrary units ± SD.

### 3.6. Gene Expression Analysis

Formalin-fixed paraffin embedded tissue was extracted with a train of alcohols and rehydrated. RNA was extracted as per Reference Gouveia et al. (2014) [53]. cDNA was synthesized using a TaqMan^®^ Reverse Transcription Reagents kit (Applied Biosystems, Foster city, CA, USA, Cat. No. N8080234), and a q-PCR was performed using the following primers: 

TP53: fw AGTGGGAATCTTCTGGGACG; rv TCTTTTGCTGGGGAGAGGAG

Bcl-2: fw TTCTTTGAGTTCGGTGGGGT; rv CAGCCTCCGTTATCCTGGAT

Caspase 9: fw GATGCTGTCCCATACCAGGA; rv TCTCGATGTACCAGGAACCG

GSK-3: fw CTCAAGGCTCTCCCCACTAG; rv GTCTTGGCCAGTCTGAGTCT

AKT: fw CAAAGGATGAAGTCGCCCAC; rv TGCAAGTACTCCAGAGCTGA

Normalization was performed using the housekeeping gen hypoxanthine-guanine phosphoribosyl transferase (HPRT): fw AGGACCTCTCGAAGTGTTGG; rv CCACTTTCGCTGATGACACA. Data were reported with respect to the control group.

### 3.7. Statistical Analyses

A one-way ANOVA by Tukey or Dunnett post hoc (*p* < 0.05) was used to perform the data analyses. The results were presented as average ± SD. Analyses were performed using the IBM SPSS Statistics for Windows (Version 22.0, IBM Corp., Armonk, NY, USA).

## 4. Conclusions

TBLF affected DMH- and AOM/DSS-induced tumorigenesis in the colon where only premalignant lesions or ACF were affected. The DMH experiment showed that TBLF decreases the incidence of premalignant lesions but no evidence of apoptotic mechanisms was observed. On the other hand, our results suggested that AOM/DSS effects were related to the induction of the Akt pathway and AiP process since an increase in cellular proliferation was observed together with high levels of p53; however, more work is required to have a better understanding of such effects. The co-administration of AOM/DSS-TBLF reversed p53 and PCNA to basal levels, suggesting an anti-proliferative effect of TBLF, and apoptosis was confirmed by the increase of caspase 9 gene expression, a decrease of Bcl-2, and the increment of caspase 3 and cytochrome-c proteins. The decrease of p-Akt after the AOM/DSS-TBLF treatment appears to be related to a blocking effect of TBLF; however, it is necessary to explore this pathway further. These results allow us to propose that TBLF induces apoptosis in the early stages of colon malignancy in a p53-independent way. Further research will focus on the signaling pathways triggered and/or altered by TBLF in colon cancer, and will propose an evaluation of the ROS-mediated effects, the extrinsic and intrinsic pathways of apoptosis, as well as other possibly triggered cell death processes to unravel the anticancer potential of TBLF as a therapeutic agent against colon cancer.
